# Stress Beyond Translation: Poxviruses and More

**DOI:** 10.3390/v8060169

**Published:** 2016-06-14

**Authors:** Jason Liem, Jia Liu

**Affiliations:** 1 Department of Microbiology and Immunology, University of Arkansas for Medical Sciences, Little Rock, Arkansas; JWLiem@uams.edu; 2 Department of Microbiology and Immunology, Center for Microbial Pathogenesis and Host Inflammatory Responses, University of Arkansas for Medical Sciences, Little Rock, Arkansas; jliu4@uams.edu

**Keywords:** poxvirus, vaccinia virus, myxoma virus, translation, stress granules, antiviral granules, antiviral stress granules, eIF2α, PKR, SAMD9

## Abstract

Poxviruses are large double-stranded DNA viruses that form viral factories in the cytoplasm of host cells. These viruses encode their own transcription machinery, but rely on host translation for protein synthesis. Thus, poxviruses have to cope with and, in most cases, reprogram host translation regulation. Granule structures, called antiviral granules (AVGs), have been observed surrounding poxvirus viral factories. AVG formation is associated with abortive poxvirus infection, and AVGs contain proteins that are typically found in stress granules (SGs). With certain mutant poxviruses lack of immunoregulatory factor(s), we can specifically examine the mechanisms that drive the formation of these structures. In fact, cytoplasmic macromolecular complexes form during many viral infections and contain sensing molecules that can help reprogram transcription. More importantly, the similarity between AVGs and cytoplasmic structures formed during RNA and DNA sensing events prompts us to reconsider the cause and consequence of these AVGs. In this review, we first summarize recent findings regarding how poxvirus manipulates host translation. Next, we compare and contrast SGs and AVGs. Finally, we review recent findings regarding RNA- and especially DNA-sensing bodies observed during viral infection.

## 1. Introduction

Poxviruses deposit and replicate their DNA exclusively in the cytoplasm, placing significant stress on the host cell. Unlike the hit-and-run tactics of RNA viruses, for example, poxviruses take over a host cell for a relatively long period of time. Although these viruses encode their own transcription machinery, they rely on the host cell for translation. Thus, it is crucial that poxviruses regulate host translation in a timely manner, ensuring a productive infection. Since mammalian cell stress responses are closely intertwined with translation control [1,2], poxvirus infection should profoundly impact host stress response pathways [3].

Poxviruses are fascinating tools with which to study host cell biology and identify intrinsic host defenses that have broad impacts on viral infection. The large, double-stranded DNA (dsDNA) poxvirus dedicates over one-third of its genome to producing proteins that evade or combat host immunity; these viral proteins are called immunoregulatory factors. Studying infections caused by poxviruses that lack immunoregulatory factors can reveal the cellular defenses that must be neutralized for a productive infection. One such defense is the formation of antiviral granules (AVGs) in the host cell. AVGs are similar to stress granules (SGs), indicating the stalled translation, but differ in a number of ways. By studying AVGs caused by poxvirus infection, we can answer new questions regarding the nature of this unique host response.

In this review, we review our current understanding of how poxvirus infection reprograms host cell translation. Since infection by poxvirus exposes the host cell to viral RNA and DNA, we discuss the similarities between AVGs and phase-dense particles that sense foreign nucleic acids. Finally, we summarize recent findings concerning the mechanisms host cells use to detect RNA and DNA within the cytoplasm.

## 2. Poxvirus Infection and Host Translation

### 2.1. Host Translation Shutoff and Differential Regulation of Host Transcripts

Due to their exclusively cytoplasmic lifestyle, poxviruses must remodel the intracellular environment of the host cell for survival. Vaccinia virus (VACV) infection is a prototypical model system that can be used to study the impact of infection on processes such as translation. Early studies showed that VACV infection in HeLa cells causes significant changes in the abundance of host transcripts that are associated with *de novo* protein synthesis. The majority of host transcripts are suppressed during the viral replication cycle, and a global shut-off of host protein synthesis can be observed [4]. However, a small fraction of host transcripts remain constant (e.g., apurinic/apyrimidinic endonuclease 2 (APEX2) and interleukin 6 signal transducer (IL-6ST)), slightly fluctuate in abundance (e.g., solute carrier family 4 member 3 (SLC4A3)), or are upregulated (e.g., pericentrin (PCNT2) and Wiskott-Aldrich syndrome protein (WASP)) [5]. WASP is important for VACV infection in the host cell [6]. However, the mechanism by which host factors such as WASP are selectively upregulated remains to be investigated.

Elevated ATP level is also crucial for VACV infection [7,8,9,10,11]. VACV specifically increases nicotinamide adenine dinucleotide dehydrogenase 4 (ND4) and cyclooxygenase-2 (COX2) protein levels, two mitochondrial proteins that function in the electron transport chain to generate ATP [12]. However, the mechanism on how synthesis of these mitochondrial proteins is regulated also remains to be elucidated.

### 2.2. Heat Shock Responses

Heat shock proteins play critical roles for cells, especially under stress conditions. Interestingly, heat shock responsive pathways are also particularly important for poxvirus infection [13,14,15,16]. How poxviruses manipulate heat shock response for their own benefit is an interesting subject. Moreover, during poxvirus infection, how heat shock proteins are differentially regulated when a global translation shut-off is triggered remains to be understood.

In fact, at least in cell culture, for an optimal productive infection, orthopoxviruses, such as VACV and monkeypox virus (MPV), require heat shock factor 1 (HSF1) [16], whose activation is often associated with stress responses [17]. The activated HSF1 functions as a transcription factor to promote transcription for genes, including Hsp90, Hsp27, and Hsp70. During VACV infection, HSF1 becomes hyperphosphorylated and is then translocated into the nucleus [16]; in this case, sustained upregulation in MAPK activity by VACV infection may indirectly promote HSF1 activation [18,19].

Intriguingly, levels of Hsp60, Hsp90, and Hsp70 mRNA and protein are not affected during VACV infection in cell culture [20,21,22,23]. VACV infection even increases the amount of 72-KDa Hsp70 protein *in vivo* [21]. Under stress conditions (e.g., heat), heat shock proteins such as HSP90 and HSP70 are preferentially translated. The mRNAs for these genes are largely excluded from stress granules (SGs), where stalled translation initiation complexes that are still associated with mRNAs are stored [2,24]. Several factors may contribute to this outcome. The lack of introns in Hsp70 mRNA causes it to be less prone to regulation by splicing factors [25], as the level of splicing factors can be drastically affected during stresses such as viral infection. The mRNAs of heat shock proteins also exit the nucleus via a unique mechanism under conditions when normal mRNA transport is blocked [26]. Moreover, a ribosomal shunting mechanism ensures the preferential translation of certain heat shock proteins such as HSP70 (due to the lack of structure within the 5’-untranslated region [5’-UTR]) [27]. However, during VACV infection, the presence of certain Hsp70 proteins, such as 72-kDa HSP70, seems to be dispensable for a productive infection in some cell types [22]. In this case, the elevated synthesis of 72-kDa HSP70 during infection may be due to the intrinsic properties of the mRNA.

HSP90, especially its ATPase activity, is functionally important for VACV to infect mammalian cells, such as RK13 (rabbit), HeLa (human), and BSC40 (monkey) [23]. HSP90 also interacts with VACV core protein 4a [23], but the biological function of this interaction is unclear. Hsp90 mRNA structure does not differ from that of other cellular mRNAs under normal temperature [28]. Under heat stress, however, the 5’-UTR of Hsp90 mRNA can resist the inhibition of 5’-cap dependent translation [28,29]. How Hsp90 remains translationally active during a VACV infection, however, remains to be investigated.

Finally, a yeast two-hybrid study identified an interaction between Hsp27/HSPB1 and VACV-WR002, C2, and I4 (TNF-α-receptor-like protein, Kelch-like protein, and ribonucleotide reductase large subunit, respectively) [30,31]. The functional consequence of these interactions, however, remains unknown.

### 2.3. Evade the Surveillance

Poxviruses must coordinate with host cellular events to effectively replicate within a host cell. To do so, these viruses employ a multifaceted strategy to evade the regulatory factors that govern host translation. This strategy includes re-organizing components of the translation initiation complex, tipping the balance of signaling pathways that regulate the translation initiation, and evading the control mechanisms of the global translation shut-down. These tactics are discussed in the later sections. Poxviruses are not the only viruses that utilize such tactics; African swine fever virus (ASFV), a large, cytoplasmic DNA virus and the sole member of *Asfarviridae*, evades host surveillance in a similar manner. First and foremost, the viral mRNA is heavily camouflaged, making it indistinguishable from cellular mRNA. For example, poxviruses encode an enzyme complex with guanylyltransferase, methyltransferase, and RNA triphosphatase activities to generate mRNA with a 5’ terminal cap of m^7^GpppN [32,33,34,35] and a 3’ poly(A) tail [36] that resembles cellular mRNA. Thus, viral protein synthesis occurs by the canonical cap-dependent mechanism and utilizes the host translation machinery. Once poxviruses enter a cell, viral early proteins are produced that reprogram the host translation machinery to deliberately boost viral protein synthesis.

### 2.4. Manipulating the Translation Machinery

**Figure 1:**
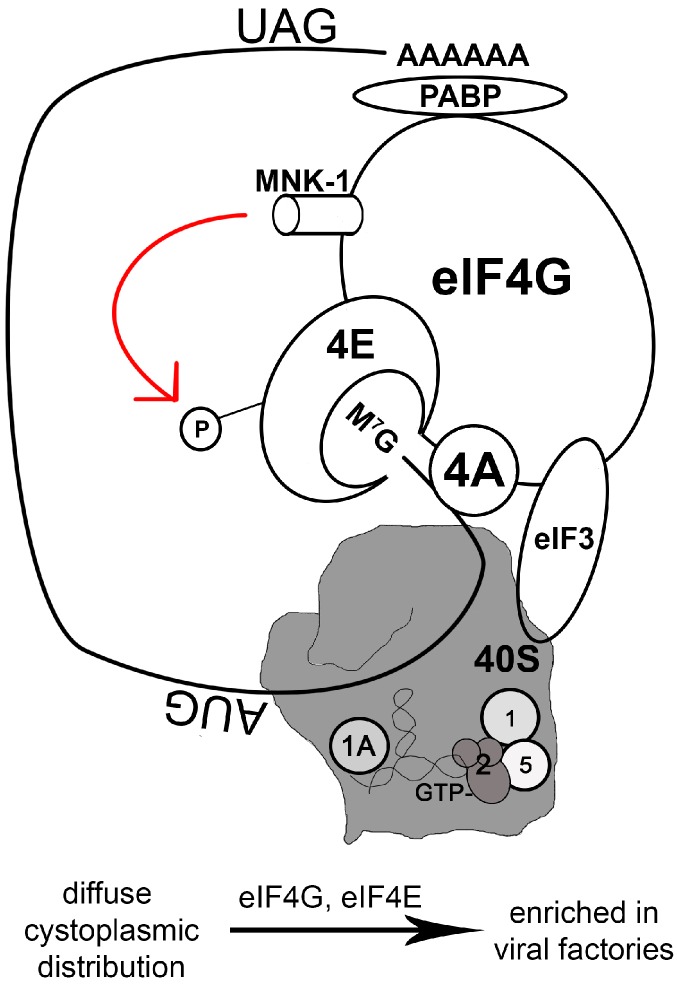
Regulation of the translation initiation complex by Mnk1 and the impact of vaccinia virus infection. For cellular or viral mRNAs containing the 7-Methylguanosine-cap structure (shown as M^7^G) on the 5’ end, recruiting host ribosomes to the mRNA is a critical event. The eukaryotic translation initiation factor 4F (eIF4F) (not shown) plays an important role in this process. eIF4F is a tripartite complex including a cap-binding subunit (eIF4E) (shown as 4E), a small RNA helicase (eIF4A) (shown as 4A), and a large scaffold protein (eIF4G) that directly interacts with the poly(A) binding protein (PABP) and a 40S-ribosome-associated protein (eIF3). The mRNA circulates by interacting with PABP at the 3’-end (depicted by a stop codon of UAG and the poly(A) tail of the mRNA) and associating with eIF4G at the 5’-end. The 43S preinitiation complex that consists of the small 40S ribosomal subunit (portrayed in a gray block at the back) and initiation factors, including eIF1 (shown as 1), eIF1A (shown as 1A), a ternary complex eIF2(α, β, γ)•GTP•met-tRNA_i_^met^ (shown as 2, GTP, and a hairpin structure, respectively), and eIF5 (shown as 5), assembles with eIF4F complex, via eIF3, to form the 48S complex. One of eIF4E kinases, Mnk1, binds to eIF4G and phosphorylates eIF4E (the red arrow represents this phosphorylation event, and the 4E connected to P represents phosphorylated eIF4E). The assembled 48S complex can then scan the mRNA for start codon (AUG). Vaccinia virus (VACV) manipulates this process by enriching the concentration of eIF4G and eIF4E in the viral factories. VACV requires Mnk1 activity for infection.

The eukaryotic translation initiation complex eIF4F is composed of an m7G-cap-binding subunit (eIF4E), a small RNA helicase (eIF4A), and a large scaffold protein (eIF4G) (Figure 1). The scaffold molecule, eIF4G, interacts with the polyadenylation [poly(A)] binding protein (PABP) that binds to the 3’-terminal region of mRNA. Thus eIF4G bridges the 5’- and 3’-termini of the mRNA via its interaction with PABP and eIF4E [37]. eIF4G also interacts with the 43S preinitiation complex (composed of eIF3, the 40S ribosomal subunit, and initiation factors such as eIF1, eIF1A, heterotrimeric eIF2(α,β,γ), and eIF5) (Figure 1) [38,39].

During a productive VACV or ASFV infection, the translation initiation complex, identified by the presence of eIF4G, eIF4E, and eIF3, is efficiently recruited to viral factories [40,41,42,43]. This compartmentalization of protein synthesis significantly favors viral protein production. Actively translated viral mRNA, however, is not all concentrated within the viral factories. For example, at 6 h post-infection in HeLa cells, F9L mRNA (the F9 protein is a membrane component of the mature VACV virion) is distributed diffusely throughout the cytoplasm [44]. In cells infected with myxoma virus (MYXV), a rabbit-specific poxvirus, components of the translation initiation complex (e.g., eIF4G) diffuse throughout the cytoplasm [45]. This may explain why the kinetics of infection is slower for MYXV than for VACV.

In stark contrast to many RNA viruses that inactivate cap-dependent translation, DNA viruses often promote the assembly of the translation initiation complex [42,43,46,47,48,49,50,51]. The inactivation of host protein synthesis by VACV is not caused by the degradation or hypophosphorylation of the eIF4F complex [4,52]. In fact, based on observations in primary cells, VACV infection can increase eIF4F protein levels [42], likely because this complex is compartmentalized during infection. During a VACV infection, the single-stranded DNA (ssDNA) binding phosphoprotein I3 brings eIF4G and eIF4G-associated proteins into close proximity with viral factories [40]. The VACV I3 protein is produced during the early to intermediate stage of the viral life cycle [53], which coincides with its binding to eIF4G at the intermediate stage of the infection [40].

**Figure 2:**
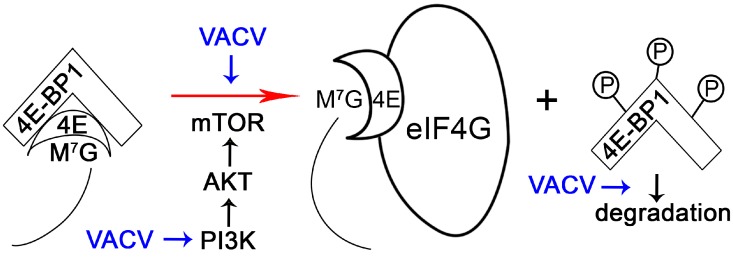
Vaccinia virus manipulates 4E-BP to favor viral protein synthesis. Mammalian 4E-BP is a translation repressor by sequestrating the cap-binding protein eIF4E. The M^7^G and the line represent the 7-Methylguanosine-cap structure and mRNA, respectively. This inhibition can be lifted through phosphorylation of 4E-BP via PI3K/AKT/mTOR axis. Vaccinia virus (VACV) infection activates PI3K (blue arrows represent the activation action), which then stimulates mTOR signaling. In turn, mTOR mediates phosphorylation of 4E-BP (shown as Ps attached to the 4E-BP1 protein), lifting the block on eIF4E (shown as 4E). In addition to enhancing phosphorylation of 4E-BP1, VACV also reduces the abundance of 4E-BP1 possibly by promoting the degradation of phosphorylated 4E-BP1.

The initiation of translation in mammalian cells is highly regulated. For example, the interaction between eIF4E and eIF4G is competitively inhibited by host cell 4E-BPs, which bind eIF4E [38]. The phosphorylation of 4E-BP1 causes the release of eIF4E that can then bind to eIF4G to assemble translation initiation complex (Figure 2). 4E-BP phosphorylation is regulated by mTOR/Akt/PI3K signaling, thus controlling translation in response to diverse stimuli [54]. It is no surprise, then, that poxviruses can manipulate PI3K/Akt signaling during infection. While MYXV uses the viral protein MT-5 to activate Akt in a PI3K-independent manner [55,56], VACV depends on PI3K activity to activate Akt [57]. Interestingly, phosphorylation of 4E-BP1 during VACV infection is dependent upon the PI3K/Akt axis, but this process is not sensitive to rapamycin [57], which targets mTOR. This suggests an alternative method of mTOR regulation (such as by association of mTOR with rictor instead of raptor [58]) or the presence of an mTOR-independent pathway to phosphorylate 4E-BP1. Regardless, phosphorylated 4E-BP1 is then subject to proteasome-dependent degradation during VACV infection [42].

eIF4E activity is also subject to regulation by a kinase called Mnk1 that is directly associated with eIF4G (Figure 1). Mnk1 promotes the phosphorylation of eIF4E, bolstering translation [59,60]. Mnk1 activity is regulated by ERK1/2 MAP kinases and stress-stimulated signals mediated by p38 [60], allowing translation to respond to environmental and cellular signals. During VACV infection, both ERK and p38 are activated, and this is associated with eIF4E activation [42]. VACV infection is severely hindered when Mnk1 is deleted or when an Mnk1 inhibitor is present [42]. Thus, not only does VACV sequester the translation initial complex, it also manipulates Mnk1 activity to boost viral protein synthesis.

Another highly-regulated component of translation initiation is eIF2α, a part of the ternary complex composed of the heterotrimeric eIF2(α,β,γ), tRNAi^Met^, and GTP. The eIF2 complex is responsible for binding tRNAi^Met^ to the 40S ribosome and for the recognition of the AUG start codon in a GTP-dependent manner. Start codon recognition leads to the hydrolysis of eIF2-bound GTP, which requires the GTPase-activating factor eIF5. Hydrolysis of GTP-eIF2 is coupled to recruitment of the large, 60S ribosome during the elongation phase of translation, when GDP-eIF2 is released for regeneration of GTP-eIF2 by eIF2B, a guanine nucleotide exchange factor. When eIF2α is phosphorylated, however, GTP-eIF2 cannot be regenerated and eIF2B is sequestrated, inhibiting translation globally. Serine/threonine kinases such as PKR phosphorylate eIF2α in response to environmental or intracellular inhibitory signals, suppressing the normal rate of translation (see review [61]). PKR is specifically targeted by viral pathogens because of its direct inhibitory effect on the viral life cycle [62,63,64]. In addition to mediating signal transduction in response to proinflammatory stimuli such as LPS and TNF-α, and participating in the IκB kinase complex, PKR also functions as a sentinel to detect dsRNA during viral infection [65].

PKR contains an N-terminal dsRNA-binding domain and a C-terminal catalytic domain; it can sense and bind to dsRNA intermediates or by-products generated during a viral infection, resulting in PKR dimerization, autophosphorylation, and kinase activation [66]. Poxviruses, without exception, develop multi-pronged strategies to evade the PKR pathway. They encode genes to produce pseudosubstrates of PKR (e.g., VACV K3L, swinepoxvirus C8L, and MYXV M156R) to divert or sequester PKR, keeping it from phosphorylating eIF2α [67,68,69,70]. The species specificity of MYXV M156 to European rabbit PKR, but not to PKR from other species, is one of the virulence determinants for pathogenesis in rabbits [71].

Since dsRNAs are generated during infection, poxviruses produce dsRNA-binding proteins, such as VACV E3 [72] and MYXV M029 [73], to prevent or delay PKR activation. When a cell is stimulated by a VACV mutant lacking E3L, PKR activates the type 1 interferon (IFN) response by modulating the activity of MDA5 [74]. Although the apparent effect of the poxvirus E3L family proteins is to bind dsRNA, these proteins possess different functions in respective viruses [75], and their modes of action *in vitro* and *in vivo* also vary [72,73,76]. Nevertheless, poxviruses utilize the E3L family of proteins to stall host translation associated with the innate immune response.

Despite the presence of PKR antagonists, poxviruses also use different strategies to minimize the production of dsRNA. The presence of two negative regulators of VACV gene expression, D9 and D10, was reported almost 10 year ago [77,78,79], but the mechanism underlying this regulation was only recently described. VACV D9 and D10 are decapping enzymes that keep the amount of viral mRNA at a minimum during infection, preventing the activation of PKR and 2’5’-oligoadenylate synthetase (OAS)/RNase L-associated RNA decay that inhibits viral protein synthesis [80,81]. In addition, VACV also takes advantage of the cellular 5’-3’ mRNA exonuclease Xrn1 to reduce the number of potential PKR and RNase L substrates [82].

Alternatively, strategic negative regulation of viral protein synthesis can be beneficial for viruses. The VACV 169 protein inhibits translation in either a cap-dependent or cap–independent manner during viral infection [83]. Infection with mutant VACV lacking the 169 gene causes enhanced lung pathology and illness in infected mice that actually promotes the clearance of these viruses [83]. Each of these strategies allows poxviruses to control the magnitude of host defense responses and deceive host innate immune surveillance, thus evading host innate and adaptive immunity.

### 2.5. Codon Usage

During translation, biased viral codon usage can have significant immunological effects. For example, when host and viral codon usage is not matched, viral protein translation decreases and immunogenicity is reduced [84,85]. VACV infection does not appear to alter either the global tRNA pool or the abundance of individual tRNA species. Instead the population of polysome-associated tRNAs is skewed based on VACV codon usage [84,86]. Despite an abundance of silenced ribosomes in the cytosol, VACV compartmentalizes translation of viral mRNA to viral factories and selectively enriches tRNAs to optimize translation. Multi-tRNA-aminoacyl synthetase (ARS) complex containing 10 ARSs becomes highly concentrated within viral factories by associating with translating ribosomes during VACV infection [86]. In addition, “free” ARSs (e.g., tyrosyl-tRNA-synthetase or YRS) that are not parts of the multi-ARS complex can also be selectively concentrated within the viral factories [86].

### 2.6. Reactive Oxygen Species (ROS)

Poxvirus infection generates reactive oxygen species (ROS) in the host cell. VACV infection leads to a switch of energy resource by fully relying on glutamine catabolism [87,88]; these changes directly influence viral protein synthesis but not viral transcription [87]. Mitochondrial β-oxidation of palmitates, products of fatty acid biosynthesis, is crucial for productive VACV infection and also provides intermediates for the tricarboxylic acid (TCA) cycle [88]. However, these processes generate superoxide anion, hydrogen superoxide, and other species of ROS. Excessive ROS production can be detrimental to host cells, but ROS also serve as signaling molecules that promote inflammation, cell proliferation, and regulate apoptosis [89,90].

Many mammalian poxviruses encode cellular Cu, Zn-superoxide dismutase (SOD) homologs that are catalytically inert [91,92,93]. Although evidently not essential for viral replication and virulence under laboratory conditions, poxvirus SODs are expressed abundantly during the late stage of infection and are packaged into virions [91,92]. While these viral SODs are capable of binding Zn, they cannot bind copper and have no catalytic activity. Instead, poxvirus SODs, such as those encoded by leporipoxviruses, form stable complexes with copper chaperones for host SODs, thus limiting the catalytic activity of host SODs [93]. This explains why the presence of viral SODs gradually decreases the activity of host SODs during infection with MYXV or Shope fibroma virus (SFV). The resulting increase in ROS may in turn inhibit apoptosis, particularly in immune cells, and promote the growth of infected cells [89]. Moreover, increases in ROS during viral infection may reprogram the immunological and pathological landscape (review by Paiva and Bozza 2014 [94]) by generating polarized Th2 cells [95] and inhibiting NK cell functions [96]. All of these factors may contribute to the tumorigenic property of some poxviruses *in vivo* (such as SFV) [93].

The effects of ROS on translation during poxvirus infection are not well characterized. However, increases in ROS appear to trigger misacylation-associated decreases in translation fidelity [97]. Translation fidelity can also be affected in response to viral infections (e.g., VACV); in these cases, methionine residues are aminoacylated to non-methionyl-tRNAs for translation [98]. The cellular and immunological implications of this phenomenon remain unexplored, but the functional alteration of gene products can certainly be expected [99].

## 3. Poxvirus Infection, Where Stress Granules and Antiviral Granules Cross Paths

### 3.1. Stress Granules

Cellular stress responses triggered by environmental stress (e.g., nutrient deprivation, oxidative stress, and endoplasmic reticulum [ER] stress) or viral infections induces the formation of cytoplasmic complexes called stress granules (SGs) that sequester host [100] and viral RNAs [44]. SGs are membraneless organelles in the cytoplasm that contain mRNA and RNA-binding proteins, and form to temporarily suppress translation when conditions are unfavorable for cell growth. Thus, SGs possess antiviral properties because they inhibit global protein synthesis.

SGs are composed of: (1) the stalled translation initiation complex (eIF3, eIF4F, the small ribosomal subunits, and PABP-1) and associated mRNA [100], (2) RNA-binding proteins that may silence translation (e.g., TIA-1, TIAR [101], and Argonaute [102]) or affect mRNA stability (e.g., endonuclease PMR1 [103]), (3) SG-nucleating factors that affect RNA metabolism (e.g., G3BP1 [104], CAPRIN1 [105]), and (4) other proteins that are recruited to SGs because they interact with SG components (e.g., TRAF2 binds to eIF4G [106]).

Many factors affect the formation and dissolution of SGs. For example, misfolded proteins are known to facilitate the formation of ribonucleoprotein granules [107], while chaperone proteins, such as HSP70, are important for the dissolution of SGs [108]. VACV infection induces the production of heat shock proteins *in vivo* [21], possibly heading off SG formation. Moreover, viruses have developed strategies to neutralize the function of SG components to prevent the sequestration of viral RNAs [109,110,111,112,113,114,115] and to avoid activating innate immune signaling [115]. Interestingly, VACV does not specifically inhibit SG formation under stress conditions (e.g., oxidative stress). It is no surprise, then, that chemicals that can stimulate SG formation have profound antiviral effects on many poxviruses, including wild-type (*wt*) VACV and monkeypox virus [44]. Thus, pharmaceuticals that potentiate SG formation may constitute effective antiviral therapies.

### 3.2. Antiviral Granules (AVGs) and the PKR/eIF2α Axis

The formation of antiviral granules (AVGs), sometimes called antiviral stress granules (avSGs) [115], can be stimulated by infection with replication-defective viruses [45,115,116]. Poxvirus infections induce the formation of AVGs around viral factories in the cytoplasm of mammalian cells [44,45,116]. While AVGs contain components common to SGs, reagents that delay or inhibit translation elongation, such as cycloheximide and emetine, cannot disassemble AVGs (as they can disassemble SGs) [45,116]. Thus, the architecture and organization of AVGs may be very different from that of SGs, and different mechanisms may drive their formation.

The formation of poxvirus AVGs is associated with abortive infection either with mutant viruses that contain deletions in genes related to host-range function (e.g., VACV E3L [116] and MYXV M062R [45]) or with a *wt* strain that causes a small number of AVGs to form spontaneously [44]. The mechanism behind the spontaneous formation of AVGs during *wt* poxvirus infection remains to be investigated. In the case of abortive infections with mutant poxviruses, AVG formation is likely related to deleted viral proteins [45,116] and their associated host targets [45,117]. However, we do not understand how AVG formation is regulated or what impact these granules have on the immunological landscape.

Poxvirus has to evade innate immune surveillance to avoid the global shutdown of translation. One major barrier is PKR activation that is associated with innate immune and stress responsive signaling, and PKR function is antagonized by the VACV E3 protein [118]. Interestingly, infection with a VACV mutant that lacks E3L induces the formation of AVGs in the cytoplasm [44,45,116]. In this case, AVGs are formed in an eIF2α-dependent manner [45,116], because AVGs do not form when murine embryonic fibroblasts expressing a dominant negative eIF2α mutation for phosphorylation (eIF2α S51A) are infected with the VACV-E3L null virus (VACV-E3LKO) [74,116].

### 3.3. Antiviral Granules and the SAMD9 Pathway

AVG formation is not entirely dependent on PKR and eIF2α, however. Sterile α motif domain-containing protein 9 (SAMD9) also affects AVG formation [45]. The SAMD9 gene is conserved in mammals, showing signatures of pathogen-driven positive selection [119]. Although it is known to possess antineoplastic properties, the biological function of SAMD9 protein remains poorly characterized.

Myxoma virus (MYXV) is a rabbit-specific poxvirus with a restricted natural host tropism for pathogenesis. Due to the oncolytic and immunotherapeutic potential of MYXV, its immunoregulatory effects in humans have attracted attention in recent years. M062R of MYXV belongs to the poxvirus host range C7L superfamily and is a functional homolog of the prototype, VACV C7L [120,121]. VACV K1L is not a member of the C7L superfamily, but possess a similar host range function and can complement the function of C7L [122]. Human SAMD9 is a target of both VACV K1L and host range C7L superfamily proteins [117,123,124], indicating that this host protein is important to the poxvirus life cycle and may play a role in host defense signaling. Importantly, abortive poxvirus infection (M062R-null MYXV or C7L and K1L double-knockout vaccinia virus [VACV-C7LK1L-DKO]) can cause human SAMD9-dependent AVG formation [45].

Although both PKR and SAMD9 are involved in AVG formation, there is no evidence that these regulatory pathways overlap. M062R-null MYXV infection of HeLa cells does not stimulate significant phosphorylation of eIF2α [45]. On the contrary, *wt* MYXV infection produces a low level of phosphorylated eIF2α during the later stage of the infection [73], possibly due to PKR activation triggered by an abundance of dsRNA. The case of eIF2α phosphorylation during infection by VACV-C7LK1L-DKO is more complex than for M062R-null MYXV infection. At 6 h post-infection with VACV-C7LK1L-DKO, very little phosphorylated eIF2α is detected in cells compared with infection by VACV E3L-null virus. However, at a later time point (e.g., 9 h post-infection), eIF2α phosphorylation can be detected in cells infected with VACV-C7LK1L-DKO. This is probably due to a lack of E3 protein synthesis and the continuous accumulation of viral dsRNAs.

SAMD9 also redistributes into SGs during stalled translation, suggesting that it may be a stress response element. It is noteworthy that the localization of SAMD9 to SGs requires treatment with a relatively high concentration of sodium arsenate over a long period of time, compared to other SG components. Regardless, the fact that SAMD9 participates in SG formation in an eIF2α phosphorylation-dependent or independent manner [45] suggests that SAMD9 may be involved in translation regulation.

In many cases, the particular organization of host proteins within SAMD9-dependent AVGs is virus specific. In M062R-null MYXV infected cells, components of the translation initiation complex (e.g., eIF4G) do not localize exclusively with G3BP1 [45], a marker of SGs and an indicator of translation suppression. Our own lab is investigating the signaling events that distinguish infection with M062R-null MYXV from infection with VACV-C7LK1L-DKO. Even though translation shutdown is thought to occur during infection with VACV-C7LK1L-DKO, SAMD9-driven formation of AVGs in M062R-null MYXV infection does not necessarily result in the complete shutdown of translation (unpublished data by Li, Nounamo, and Liu).

### 3.4. Antiviral Granules and Innate Sensing Molecules

Intriguingly, sensing molecules and antiviral factors, such as RIG-I, MDA-5, PKR, OAS, and RNase L, are detected within AVGs formed during infection with various viruses [115], and some of these factors can be detected in AVGs generated during poxvirus infection (unpublished data by J. Liu). In certain cases, these interactions have been confirmed via co-immunoprecipitation [74,125]. These sensing molecules appear to enhance type I IFN expression via IRF3 [74,115], and the presence of viral RNA seems to coincide with the AVG-associated co-localization of sensing molecules and IFN activation. This co-localization can be observed during infection with both RNA [115] DNA viruses [45]. Both polyinosinic-polycytidylic acid [poly(I:C)] and viral RNA are able to stimulate the granule formation in a PKR-dependent manner. The composition of these granule structures varies, however. For example, SAMD9, which is antagonized by MYXV M062, VACV C7, and VACV K1, does not localize to poly(I:C)-stimulated granules (unpublished data by J. Liu). Instead, SAMD9 determines the organization of AVGs formed during infection with M062R-null MYXV and the VACV C7L and K1L double-knockout virus [45]. In fact, in some cell lines, the presence of C7L and K1L provides resistance to type I IFN and the IRF1-stimulated antiviral stage during VACV infection [126]. In addition to type I IFN activation, we expect diverse signaling events can be initiated during the organization of AVGs as those observed in SGs [127].

In summary, it is important to investigate the function and regulation of AVGs to better understand host-intrinsic defenses against viral pathogens at a cellular level. We can utilize poxvirus immunoregulatory factors (e.g., MYXV M062) as tools to probe and dissect the molecular mechanisms that link AVGs and signal transduction events that orchestrate antiviral effects. We should also consider whether AVGs serve different functions depending on the presence of particular stimuli (e.g., the presence of exclusively foreign RNA or DNA in the cytoplasm). Thus, in the following discussion, we consider the biological functions of poxvirus-stimulated granules in light of their similarity to other RNA- and DNA-sensing bodies that are generated during the innate immune response to viral infections.

## 4. SGs and RNA-stimulated Antiviral Stress Granules as Signaling Hubs

SG and AVG formation trigger different downstream signaling events depending on the type of viral infection. The term “antiviral stress granule” (avSG) is often inter-changeable with AVG, but avSG is typically used in cases where RNA viruses manipulate the formation of SG-like structures. The assembly of avSGs during viral infection may enhance the innate immune response.

The N-terminal domain of PKR is important for avSG-associated type I IFN activation [115], while the kinase domain is dispensable [128,129]. Infection with a mutant influenza A virus (IAV) that lacks non-structural protein 1 (NS1) causes the formation of avSGs [115]. This 2012 study by Onomoto *et al.* [115] links the recruitment of sensing molecules such as MDA5 and RIG-I to avSGs with the activation of IFN signaling. A separate study investigated the effect of IAV NS1 on granule formation and suggested that these granules serve as signal transduction hubs, but the activation of RNA sensor(s) is not essential for PKR-dependent SG formation [130].

Encephalomyocarditis virus (EMCV) infection causes transient SG formation that is quickly resolved by the viral protein 3C protease via its cleavage of G3BP1 [125]. Mutant G3BP1 that is resistant to cleavage sustains the formation of avSGs, resulting in the elevated expression of innate immune cytokines and type I IFN [125]. EMCV leader (L) protein, on the other hand, inhibits avSG formation in a PKR-dependent manner and can also suppress type I IFN production [131]. At the time of avSG formation where phosphorylated PKR interacts with G3BP1, MDA5 is recruited to granules containing G3BP1, TIAR, and dsRNA during EMCV infection. The enrichment of MDA5, and possibly its substrates, in these avSGs optimizes signal transduction to upregulate cytokine expression [125]. In a separate study, however, MDA5 pathway activation did not trigger granule formation [128]. Moreover, MDA5 localization into avSGs is dispensable for type 1I IFN expression. Thus, avSG formation can have various consequences, and the factors that regulate avSG formation appear to be particularly complex.

Finally, infection with Sendai virus strain Cantell generates RNA species that stimulate the formation of two kinds of cytoplasmic granule structures. In addition to avSG-like complexes, an avSG–independent granule structure forms but lacks SG components and sensor molecules [132]. Both granules harbor RNA species that are potent type I IFN inducers. Nevertheless, this evidence suggests that a somewhat convoluted mechanism governs RNA surveillance within the cytosolic compartment.

Interestingly, overexpressing SG components triggers innate immune signaling and associated transcription remodeling. For example, ectopic expression of G3BP1 promotes NF-κB- and JNK-associated transcriptional activation [127]. In this case, inactivated PKR is recruited to SGs for activation in order to conduct antiviral activities. These conclusions are supported by the evidence that mostly inactivated PKRs were recruited to SGs formed after G3BP1 overexpression [127]. Thus, G3BP1 is an important factor associated with the signaling events that take place within SGs.

In the event of an RNA virus infection, the formation of cytoplasmic granules coincides with the antiviral response. Although the exact triggers of antiviral signaling may vary for different RNA viruses, these cytoplasmic granules serve as a regulatory hub from which to organize an appropriate response. As a common component of SGs, G3BP1 recruits other signaling factors that induce transcription remodeling. Among these factors and depending on the pathogen, the roles of innate immune sensors (e.g., PKR, MDA5, or RIG-I) may have to be individually characterized. The precise mechanism by which these distinct granules (avSG-like or –independent) are regulated remains to be determined.

## 5. Cytoplasmic Bodies Containing Viral DNA: Signaling Beyond Translation

The AVGs formed during poxvirus infection contain viral DNA, but it is not known if this viral DNA activates unique antiviral signaling pathways. It is noteworthy, however, that these cytoplasmic complexes may not regulate translation directly.

Cytosolic DNA presents a dangerous signal, and mammalian cells have developed specialized sentinels to patrol cytoplasmic compartments and resolve crises. Since poxviruses deposit and replicate their genomic DNA in distinct cytoplasmic compartments, these viruses are useful tools with which to study DNA-stimulated immune sensing and signal transduction. For example, VACV C16 protein binds to DNA-dependent protein kinase (DNA-PK) to prevent DNA sensing in a cell-type dependent manner [133]. Thus, the potential link between the stimulated antiviral state and the presence of cytosolic DNA during abortive poxvirus infection encourages a closer examination of the DNA-sensing mechanisms used by mammalian cells. Much of the data on how these DNA-containing granule structures stimulate a type I IFN response came from studies of other viruses [134,135,136,137,138,139,140,141].

### 5.1. DNA-Sensing Bodies Containing IFN γ-inducible Protein 16 (IFI16)

Herpesviruses typically deposit their DNA into the host cell nucleus. In some cell types, however, herpesvirus particles are ubiquitinated and degraded by the proteasome, exposing viral DNA to the cytoplasmic compartment [142]. IFN γ-inducible protein 16 (IFI16) is a cellular sensor for transfected DNA and herpes simplex virus type 1 (HSV-1); IFI16, via activation of STING, coordinates IRF3 and NF-κB signaling to induce type I IFN and the expression of other cytokines [135].

Human IFI16 and murine IFI204 (p204) belong to the PYHIN (pyrin and HIN domain-containing) protein family, and both bind DNA with a HIN200 domain. Although IFI16 is predominantly localized to the nucleus [143], acetylation of IFI16 at nuclear-localization signal motifs lead to its retention in the cytoplasm, allowing it to sense HSV DNA [139]. In this case, IFI16 associates with cytoplasmic viral HSV-1 DNA [142]; these cytoplasmic sensing bodies are similar in function to AVGs, but do not contain RNA or translation machinery.

These IFI16 DNA-sensing bodies have been initially characterized. First, IFI16 is present in the cytoplasm in a cell-type dependent manner (e.g., in macrophages and lymphocytes), probably due to the presence of specific acetyltransferases or deacetylases in the cytoplasm. Next, along with HSV DNA and IFI16, STING, the sensing adaptor molecule, and some proteasome components are co-localized [142]. Upon DNA sensing, STING is redistributed into discrete cytoplasmic foci [144]. Moreover, STING translocation from the endoplasmic reticulum (ER) to perinuclear vesicles relies on Sec5 and the translocon-associated protein complex (TRAP) [144] (Sec5 is a component of the octameric exocyst complex [145] and TRAP is a component of ER-associated translocation machinery [146,147]). STING interacts with TBK1 to trigger type I IFN. TBK1, on the other hand, is regulated by essential autophagy proteins (e.g., Atg9a) [148], and dsDNA is a sufficient stimulus to redistribute TBK1 to the punctate structures [148].

These findings suggest the presence of a previously uncharacterized DNA-containing cellular entity that organizes selective proteasome activity, enriches sensing, and orchestrates adaptor molecules to respond to viral infection. To further complicate the matter, however, other antimicrobial-signaling pathways are channeled through this DNA-sensing. For example, cytosolic DNA sensing by IFI16 also activates inflammasomes, at least in CD4^+^ T cells [136,137,138,149].

### 5.2. Other DNA-Sensing Bodies

Numerous DNA sensors have been identified in the mammalian cell. In resting, non-monocytic cells [150], Ku70, Ku80, and the catalytic subunit of DNA-PK (all of which make up the DNA-PK complex) bind to cytosolic DNA to form punctate structures. TBK1 localizes to these structures to trigger IRF3-dependent innate immune responses [151]. Furthermore, seemingly redundant DNA-sensing molecules utilize STING as a common adaptor to trigger the IFN response. For example, intracellular sensing of foreign DNA in conventional dendritic cells (cDC) by DDX41, a helicase, via STING has been reported [152]. Meiotic recombination 11 homolog A (MRE11) coordinates with RAD50 to mediate DNA sensing upstream of STING, as well [153]. Cyclic GMP-AMP synthase (cGAS), another DNA sensor, forms complexes with foreign DNA in macrophages [141,154]. Intriguingly, cGAS and IFI16 seem to interact, and this interaction appears to stabilize IFI16, preventing its proteasome-associated degradation [141]. Finally, p53 and HIN 200 family members, including p202 and antaxia-telangiectasia mutated kinase (ATM), can serve as DNA sensors in the cytoplasm to trigger apoptosis [155] and regulate caspase activation [156,157], respectively.

Overall, it is not clear why so many different DNA sensors exist for cytosolic surveillance; although these sensing molecules vary by cell type, this alone does not account for their diversity. We should also examine the differences in target recognition among individual cytosolic DNA sensors. Moreover, whether these seemingly redundant DNA sensors are regulated by the organization of cytoplasmic granule structures remains to be investigated. In the case of herpesvirus DNA, a surveillance system composed of proteasomes seems to be in place to digest the viral capsid and coordinate subsequent DNA sensing [142]. These series of events then influence signal transductions that activate distinct gene expression program. In professional immune cells such as macrophages and cDCs, these specialized DNA sensing mechanisms affect not only the innate response, but they also potentiate the activation of adaptive immunity [158]. Overall, these data raise new questions regarding the biological relevance of cytoplasmic DNA-associated sensing bodies in terms of pathogenesis and pro-inflammatory or autoimmune conditions.

## 6. Concluding Remarks

Immunoregulatory factors produced by poxviruses are novel tools that can help us understand host immune defense mechanisms. Mutant poxviruses that lack immunomodulatory genes allow us to discover novel cellular responses that are important for intrinsic immunity and overall cell biology. Poxviruses reprogram host cell translation to benefit the viral life cycle, but the dsDNA and RNA intermediates (or RNA byproducts) generated during poxvirus infection present danger signals to the host cell. Thus, poxviruses use viral factors to neutralize host defense molecules that are detrimental to the viral life cycle.

The observation that poxvirus infection induces the formation of AVGs surrounding viral factories raises some key questions: are these granular structures indicative of unique host defenses associated with translation control? What is the decision point for a host cell to generate AVGs? It appears that either the PKR/eIF2α phosphorylation axis or the SAMD9 pathway controls the AVG formation, but there may be additional regulatory mechanisms governing this process.

It will be intriguing to see whether the SAMD9 pathway regulates translation in some way. Recent progress in the study of cellular RNA- and DNA-sensing reveals a dynamic host surveillance system in the cytoplasm. These unique entities, including poxvirus-stimulated granules and sensing bodies, prompt us to expand our view of how these structures are assembled. Thus, poxvirus is an invaluable tool with which we can examine host regulatory mechanisms that function beyond translation.

## References

[1] Anderson P., Kedersha N. (2002). Stressful initiations. J. Cell Sci..

[2] Kedersha N., Anderson P. (2002). Stress granules: Sites of mRNA triage that regulate mRNA stability and translatability. Biochem. Soc. Trans..

[3] Walsh D., Mohr I. (2011). Viral subversion of the host protein synthesis machinery. Nat. Rev. Microbio..

[4] Schnierle B.S., Moss B. (1992). Vaccinia virus-mediated inhibition of host protein synthesis involves neither degradation nor underphosphorylation of components of the cap-binding eukaryotic translation initiation factor complex eIF-4F. Virology.

[5] Guerra S., Lopez-Fernandez L.A., Pascual-Montano A., Munoz M., Harshman K., Esteban M. (2003). Cellular gene expression survey of vaccinia virus infection of human HeLa cells. J. Virol..

[6] Guerra S., Aracil M., Conde R., Bernad A., Esteban M. (2005). Wiskott-aldrich syndrome protein is needed for vaccinia virus pathogenesis. J. Virol..

[7] Gershowitz A., Boone R.F., Moss B. (1978). Multiple roles for ATP in the synthesis and processing of mRNA by vaccinia virus: Specific inhibitory effects of adenosine (beta,gamma-imido) triphosphate. J. Virol..

[8] Shuman S., Spencer E., Furneaux H., Hurwitz J. (1980). The role of ATP in *in vitro* vaccinia virus RNA synthesis effects of AMP-PNP and ATP gamma S. J. Biol. Chem..

[9] Foglesong P.D., Bauer W.R. (1984). Effects of ATP and inhibitory factors on the activity of vaccinia virus type i topoisomerase. J. Virol..

[10] Deng L., Shuman S. (1998). Vaccinia NPH-I, a dexh-box ATPase, is the energy coupling factor for mRNA transcription termination. Genes Dev..

[11] Boyle K.A., Arps L., Traktman P. (2007). Biochemical and genetic analysis of the vaccinia virus d5 protein: Multimerization-dependent ATPase activity is required to support viral DNA replication. J. Virol..

[12] Chang C.W., Li H.C., Hsu C.F., Chang C.Y., Lo S.Y. (2009). Increased ATP generation in the host cell is required for efficient vaccinia virus production. J. Biomed. Sci..

[13] Mercer J., Snijder B., Sacher R., Burkard C., Bleck C.K., Stahlberg H., Pelkmans L., Helenius A. (2012). RNAi screening reveals proteasome- and Cullin3-dependent stages in vaccinia virus infection. Cell Rep..

[14] Sivan G., Martin S.E., Myers T.G., Buehler E., Szymczyk K.H., Ormanoglu P., Moss B. (2013). Human genome-wide RNAi screen reveals a role for nuclear pore proteins in poxvirus morphogenesis. Proc. Natl. Acad. Sci. USA.

[15] Teferi W.M., Dodd K., Maranchuk R., Favis N., Evans D.H. (2013). A whole-genome RNA interference screen for human cell factors affecting myxoma virus replication. J. Virol..

[16] Filone C.M., Caballero I.S., Dower K., Mendillo M.L., Cowley G.S., Santagata S., Rozelle D.K., Yen J., Rubins K.H., Hacohen N. (2014). The master regulator of the cellular stress response (HSF1) is critical for orthopoxvirus infection. PLoS Pathog..

[17] Guettouche T., Boellmann F., Lane W.S., Voellmy R. (2005). Analysis of phosphorylation of human heat shock factor 1 in cells experiencing a stress. BMC Biochem..

[18] de Magalhaes J.C., Andrade A.A., Silva P.N., Sousa L.P., Ropert C., Ferreira P.C., Kroon E.G., Gazzinelli R.T., Bonjardim C.A. (2001). A mitogenic signal triggered at an early stage of vaccinia virus infection: Implication of mek/erk and protein kinase A in virus multiplication. J. Biol. Chem..

[19] Andrade A.A., Silva P.N., Pereira A.C., De Sousa L.P., Ferreira P.C., Gazzinelli R.T., Kroon E.G., Ropert C., Bonjardim C.A. (2004). The vaccinia virus-stimulated mitogen-activated protein kinase (MAPK) pathway is required for virus multiplication. Biochem. J..

[20] Jindal S., Young R.A. (1992). Vaccinia virus infection induces a stress response that leads to association of Hsp70 with viral proteins. J. Virol..

[21] Sedger L., Ruby J. (1994). Heat shock response to vaccinia virus infection. J. Virol..

[22] Sedger L., Ramshaw I., Condie A., Medveczky J., Braithwaite A., Ruby J. (1996). Vaccinia virus replication is independent of cellular Hsp72 expression which is induced during virus infection. Virology.

[23] Hung J.J., Chung C.S., Chang W. (2002). Molecular chaperone Hsp90 is important for vaccinia virus growth in cells. J. Virol..

[24] Stohr N., Lederer M., Reinke C., Meyer S., Hatzfeld M., Singer R.H., Huttelmaier S. (2006). Zbp1 regulates mRNA stability during cellular stress. J. Cell Biol..

[25] Baguet A., Degot S., Cougot N., Bertrand E., Chenard M.P., Wendling C., Kessler P., Le Hir H., Rio M.C., Tomasetto C. (2007). The exon-junction-complex-component metastatic lymph node 51 functions in stress-granule assembly. J. Cell Sci..

[26] Yost H.J., Petersen R.B., Lindquist S. (1990). RNA metabolism: Strategies for regulation in the heat shock response. Trends Genet. TIG.

[27] Rubtsova M.P., Sizova D.V., Dmitriev S.E., Ivanov D.S., Prassolov V.S., Shatsky I.N. (2003). Distinctive properties of the 5'-untranslated region of human Hsp70 mRNA. J. Biol. Chem..

[28] Ahmed R., Duncan R.F. (2004). Translational regulation of Hsp90 mRNA. Aug-proximal 5'-untranslated region elements essential for preferential heat shock translation. J. Biol. Chem..

[29] Duncan R.F. (2008). Rapamycin conditionally inhibits Hsp90 but not Hsp70 mRNA translation in drosophila: Implications for the mechanisms of Hsp mRNA translation. Cell Stress Chaperones.

[30] Van Vliet K., Mohamed M.R., Zhang L., Villa N.Y., Werden S.J., Liu J., McFadden G. (2009). Poxvirus proteomics and virus-host protein interactions. Microbiol. Mol. Biol. Rev. MMBR.

[31] Zhang L., Villa N.Y., Rahman M.M., Smallwood S., Shattuck D., Neff C., Dufford M., Lanchbury J.S., Labaer J., McFadden G. (2009). Analysis of vaccinia virus-host protein-protein interactions: Validations of yeast two-hybrid screenings. J. Proteome Res..

[32] Ensinger M.J., Martin S.A., Paoletti E., Moss B. (1975). Modification of the 5′-terminus of mRNA by soluble guanylyl and methyl transferases from vaccinia virus. Proc. Natl. Acad. Sci. USA.

[33] Martin S.A., Moss B. (1975). Modification of RNA by mRNA guanylyltransferase and mRNA (guanine-7-) methyltransferase from vaccinia virions. J. Biol. Chem..

[34] Boone R.F., Moss B. (1977). Methylated 5'-terminal sequences of vaccinia virus mRNA species made *in vivo* at early and late times after infection. Virology.

[35] Venkatesan S., Gershowitz A., Moss B. (1980). Modification of the 5' end of mRNA. Association of RNA triphosphatase with the RNA guanylyltransferase-RNA (guanine-7-)methyltransferase complex from vaccinia virus. J. Biol. Chem..

[36] Atherton K.T., Darby G. (1974). Patterns of transcription of messengers containing poly A in vaccinia virus-infected cells. J. Gen. Virol..

[37] Wells S.E., Hillner P.E., Vale R.D., Sachs A.B. (1998). Circularization of mRNA by eukaryotic translation initiation factors. Mol. Cell.

[38] Pause A., Belsham G.J., Gingras A.C., Donze O., Lin T.A., Lawrence J.C., Sonenberg N. (1994). Insulin-dependent stimulation of protein synthesis by phosphorylation of a regulator of 5'-cap function. Nature.

[39] Reineke L.C., Lloyd R.E. (2011). Animal virus schemes for translation dominance. Curr. Opin. Virol..

[40] Zaborowska I., Kellner K., Henry M., Meleady P., Walsh D. (2012). Recruitment of host translation initiation factor eif4g by the vaccinia virus ssDNA-binding protein I3. Virology.

[41] Katsafanas G.C., Moss B. (2007). Colocalization of transcription and translation within cytoplasmic poxvirus factories coordinates viral expression and subjugates host functions. Cell Host Microbe.

[42] Walsh D., Arias C., Perez C., Halladin D., Escandon M., Ueda T., Watanabe-Fukunaga R., Fukunaga R., Mohr I. (2008). Eukaryotic translation initiation factor 4f architectural alterations accompany translation initiation factor redistribution in poxvirus-infected cells. Mol. Cell. Biol..

[43] Castello A., Quintas A., Sanchez E.G., Sabina P., Nogal M., Carrasco L., Revilla Y. (2009). Regulation of host translational machinery by african swine fever virus. PLoS Pathog..

[44] Rozelle D.K., Filone C.M., Kedersha N., Connor J.H. (2014). Activation of stress response pathways promotes formation of antiviral granules and restricts virus replication. Mol. Cell. Biol..

[45] Liu J., McFadden G. (2015). SAMD9 is an innate antiviral host factor with stress response properties that can be antagonized by poxviruses. J. Virol..

[46] Arias C., Walsh D., Harbell J., Wilson A.C., Mohr I. (2009). Activation of host translational control pathways by a viral developmental switch. PLoS Pathog..

[47] Kudchodkar S.B., Yu Y., Maguire T.G., Alwine J.C. (2004). Human cytomegalovirus infection induces rapamycin-insensitive phosphorylation of downstream effectors of mtor kinase. J. Virol..

[48] McMahon R., Zaborowska I., Walsh D. (2011). Noncytotoxic inhibition of viral infection through eif4f-independent suppression of translation by 4EGI-1. J. Virol..

[49] O’Shea C., Klupsch K., Choi S., Bagus B., Soria C., Shen J., McCormick F., Stokoe D. (2005). Adenoviral proteins mimic nutrient/growth signals to activate the mtor pathway for viral replication. EMBO J..

[50] Moorman N.J., Shenk T. (2010). Rapamycin-resistant mtorc1 kinase activity is required for herpesvirus replication. J. Virol..

[51] Walsh D. (2010). Manipulation of the host translation initiation complex eif4f by DNA viruses. Biochem. Soc. Trans..

[52] Gierman T.M., Frederickson R.M., Sonenberg N., Pickup D.J. (1992). The eukaryotic translation initiation factor 4e is not modified during the course of vaccinia virus replication. Virology.

[53] Rochester S.C., Traktman P. (1998). Characterization of the single-stranded DNA binding protein encoded by the vaccinia virus I3 gene. J. Virol..

[54] Gingras A.C., Gygi S.P., Raught B., Polakiewicz R.D., Abraham R.T., Hoekstra M.F., Aebersold R., Sonenberg N. (1999). Regulation of 4e-bp1 phosphorylation: A novel two-step mechanism. Genes Dev..

[55] Wang G., Barrett J.W., Stanford M., Werden S.J., Johnston J.B., Gao X., Sun M., Cheng J.Q., McFadden G. (2006). Infection of human cancer cells with myxoma virus requires akt activation via interaction with a viral ankyrin-repeat host range factor. Proc. Natl. Acad. Sci. USA.

[56] Stanford M.M., Barrett J.W., Nazarian S.H., Werden S., McFadden G. (2007). Oncolytic virotherapy synergism with signaling inhibitors: Rapamycin increases myxoma virus tropism for human tumor cells. J. Virol..

[57] Zaborowska I., Walsh D. (2009). PI3k signaling regulates rapamycin-insensitive translation initiation complex formation in vaccinia virus-infected cells. J. Virol..

[58] Sarbassov D.D., Ali S.M., Kim D.H., Guertin D.A., Latek R.R., Erdjument-Bromage H., Tempst P., Sabatini D.M. (2004). Rictor, a novel binding partner of mtor, defines a rapamycin-insensitive and raptor-independent pathway that regulates the cytoskeleton. Curr. Biol. CB.

[59] Fukunaga R., Hunter T. (1997). Mnk1, a new map kinase-activated protein kinase, isolated by a novel expression screening method for identifying protein kinase substrates. EMBO J..

[60] Waskiewicz A.J., Flynn A., Proud C.G., Cooper J.A. (1997). Mitogen-activated protein kinases activate the serine/threonine kinases mnk1 and mnk2. EMBO J..

[61] Anderson P., Kedersha N. (2002). Visibly stressed: The role of eif2, TIA-1, and stress granules in protein translation. Cell Stress Chaperones.

[62] Dar A.C., Dever T.E., Sicheri F. (2005). Higher-order substrate recognition of eIF2alpha by the RNA-dependent protein kinase PKR. Cell.

[63] Dey M., Cao C., Dar A.C., Tamura T., Ozato K., Sicheri F., Dever T.E. (2005). Mechanistic link between PKR dimerization, autophosphorylation, and eIF2alpha substrate recognition. Cell.

[64] Daugherty M.D., Malik H.S. (2012). Rules of engagement: Molecular insights from host-virus arms races. Ann. Rev. Genet..

[65] Williams B.R. (2001). Signal integration via PKR. Science’s STKE: Signal transduction knowledge environment.

[66] Garcia M.A., Meurs E.F., Esteban M. (2007). The dsRNA protein kinase PKR: Virus and cell control. Biochimie.

[67] Kawagishi-Kobayashi M., Cao C., Lu J., Ozato K., Dever T.E. (2000). Pseudosubstrate inhibition of protein kinase PKR by swine pox virus c8l gene product. Virology.

[68] Elde N.C., Child S.J., Geballe A.P., Malik H.S. (2009). Protein kinase r reveals an evolutionary model for defeating viral mimicry. Nature.

[69] Rothenburg S., Seo E.J., Gibbs J.S., Dever T.E., Dittmar K. (2009). Rapid evolution of protein kinase PKR alters sensitivity to viral inhibitors. Nat. Struct. Mol. Biol..

[70] Ramelot T.A., Cort J.R., Yee A.A., Liu F., Goshe M.B., Edwards A.M., Smith R.D., Arrowsmith C.H., Dever T.E., Kennedy M.A. (2002). Myxoma virus immunomodulatory protein m156r is a structural mimic of eukaryotic translation initiation factor eIF2alpha. J. Mol. Biol..

[71] Peng C., Haller S.L., Rahman M.M., McFadden G., Rothenburg S. (2016). Myxoma virus m156 is a specific inhibitor of rabbit PKR but contains a loss-of-function mutation in australian virus isolates. Proc. Natl. Acad. Sci. USA.

[72] Chang H.W., Watson J.C., Jacobs B.L. (1992). The e3l gene of vaccinia virus encodes an inhibitor of the interferon-induced, double-stranded RNA-dependent protein kinase. Proc. Natl. Acad. Sci. USA.

[73] Rahman M.M., Liu J., Chan W.M., Rothenburg S., McFadden G. (2013). Myxoma virus protein m029 is a dual function immunomodulator that inhibits PKR and also conscripts rha/dhx9 to promote expanded host tropism and viral replication. PLoS Pathog..

[74] Pham A.M., Santa Maria F.G., Lahiri T., Friedman E., Marie I.J., Levy D.E. (2016). PKR transduces mda5-dependent signals for type i ifn induction. PLoS Pathog..

[75] Vijaysri S., Talasela L., Mercer A.A., McInnes C.J., Jacobs B.L., Langland J.O. (2003). The orf virus e3l homologue is able to complement deletion of the vaccinia virus e3l gene *in vitro* but not *in vivo*. Virology.

[76] Brandt T., Heck M.C., Vijaysri S., Jentarra G.M., Cameron J.M., Jacobs B.L. (2005). The n-terminal domain of the vaccinia virus E3l-protein is required for neurovirulence, but not induction of a protective immune response. Virology.

[77] Parrish S., Moss B. (2006). Characterization of a vaccinia virus mutant with a deletion of the D10r gene encoding a putative negative regulator of gene expression. J. Virol..

[78] Parrish S., Moss B. (2007). Characterization of a second vaccinia virus mRNA-decapping enzyme conserved in poxviruses. J. Virol..

[79] Parrish S., Resch W., Moss B. (2007). Vaccinia virus D10 protein has mRNA decapping activity, providing a mechanism for control of host and viral gene expression. Proc. Natl. Acad. Sci. USA.

[80] Liu S.W., Wyatt L.S., Orandle M.S., Minai M., Moss B. (2014). The D10 decapping enzyme of vaccinia virus contributes to decay of cellular and viral mRNAs and to virulence in mice. J. Virol..

[81] Liu S.W., Katsafanas G.C., Liu R., Wyatt L.S., Moss B. (2015). Poxvirus decapping enzymes enhance virulence by preventing the accumulation of dsRNA and the induction of innate antiviral responses. Cell Host Microbe.

[82] Burgess H.M., Mohr I. (2015). Cellular 5′-3′ mRNA exonuclease xrn1 controls double-stranded RNA accumulation and anti-viral responses. Cell Host Microbe.

[83] Strnadova P., Ren H., Valentine R., Mazzon M., Sweeney T.R., Brierley I., Smith G.L. (2015). Inhibition of translation initiation by protein 169: A vaccinia virus strategy to suppress innate and adaptive immunity and alter virus virulence. PLoS Pathog..

[84] Pavon-Eternod M., David A., Dittmar K., Berglund P., Pan T., Bennink J.R., Yewdell J.W. (2013). Vaccinia and influenza a viruses select rather than adjust tRNAs to optimize translation. Nucl. Acids Res..

[85] Martinez O., Miranda E., Ramirez M., Santos S., Rivera C., Vazquez L., Sanchez T., Tremblay R.L., Rios-Olivares E., Otero M. (2015). Immunomodulator-based enhancement of anti smallpox immune responses. PLoS ONE.

[86] David A., Netzer N., Strader M.B., Das S.R., Chen C.Y., Gibbs J., Pierre P., Bennink J.R., Yewdell J.W. (2011). RNA binding targets aminoacyl-tRNA synthetases to translating ribosomes. J. Biol. Chem..

[87] Fontaine K.A., Camarda R., Lagunoff M. (2014). Vaccinia virus requires glutamine but not glucose for efficient replication. J. Virol..

[88] Greseth M.D., Traktman P. (2014). De novo fatty acid biosynthesis contributes significantly to establishment of a bioenergetically favorable environment for vaccinia virus infection. PLoS Pathog..

[89] Teoh M.L., Turner P.V., Evans D.H. (2005). Tumorigenic poxviruses up-regulate intracellular superoxide to inhibit apoptosis and promote cell proliferation. J. Virol..

[90] D’Autreaux B., Toledano M.B. (2007). Ros as signalling molecules: Mechanisms that generate specificity in ros homeostasis. Nat. Rev. Mol. Cell Biol..

[91] Almazan F., Tscharke D.C., Smith G.L. (2001). The vaccinia virus superoxide dismutase-like protein (a45r) is a virion component that is nonessential for virus replication. J. Virol..

[92] Cao J.X., Teoh M.L., Moon M., McFadden G., Evans D.H. (2002). Leporipoxvirus cu-zn superoxide dismutase homologs inhibit cellular superoxide dismutase, but are not essential for virus replication or virulence. Virology.

[93] Teoh M.L., Walasek P.J., Evans D.H. (2003). Leporipoxvirus cu,zn-superoxide dismutase (sod) homologs are catalytically inert decoy proteins that bind copper chaperone for sod. J. Biol. Chem..

[94] Paiva C.N., Bozza M.T. (2014). Are reactive oxygen species always detrimental to pathogens?. Antioxid. Redox Signal..

[95] Gilmour M.I. (2012). Influence of air pollutants on allergic sensitization: The paradox of increased allergies and decreased resistance to infection. Toxicol. Pathol..

[96] Fortin C., Huang X., Yang Y. (2012). Nk cell response to vaccinia virus is regulated by myeloid-derived suppressor cells. J. Immunol..

[97] Lee J.Y., Kim D.G., Kim B.G., Yang W.S., Hong J., Kang T., Oh Y.S., Kim K.R., Han B.W., Hwang B.J. (2014). Promiscuous methionyl-tRNA synthetase mediates adaptive mistranslation to protect cells against oxidative stress. J. Cell Sci..

[98] Netzer N., Goodenbour J.M., David A., Dittmar K.A., Jones R.B., Schneider J.R., Boone D., Eves E.M., Rosner M.R., Gibbs J.S. (2009). Innate immune and chemically triggered oxidative stress modifies translational fidelity. Nature.

[99] Wang X., Pan T. (2015). Methionine mistranslation bypasses the restraint of the genetic code to generate mutant proteins with distinct activities. PLoS Genet..

[100] Anderson P., Kedersha N. (2008). Stress granules: The tao of RNA triage. Trends Biochem. Sci..

[101] Kedersha N.L., Gupta M., Li W., Miller I., Anderson P. (1999). RNA-binding proteins TIA-1 and tiar link the phosphorylation of eif-2 alpha to the assembly of mammalian stress granules. J. Cell Biol..

[102] Leung A.K., Calabrese J.M., Sharp P.A. (2006). Quantitative analysis of argonaute protein reveals microRNA-dependent localization to stress granules. Proc. Natl. Acad. Sci. USA.

[103] Yang F., Peng Y., Murray E.L., Otsuka Y., Kedersha N., Schoenberg D.R. (2006). Polysome-bound endonuclease pmr1 is targeted to stress granules via stress-specific binding to TIA-1. Mol. Cell. Biol..

[104] Tourriere H., Chebli K., Zekri L., Courselaud B., Blanchard J.M., Bertrand E., Tazi J. (2003). The rasgap-associated endoribonuclease G3BP assembles stress granules. J. Cell Biol..

[105] Solomon S., Xu Y., Wang B., David M.D., Schubert P., Kennedy D., Schrader J.W. (2007). Distinct structural features of caprin-1 mediate its interaction with G3BP-1 and its induction of phosphorylation of eukaryotic translation initiation factor 2alpha, entry to cytoplasmic stress granules, and selective interaction with a subset of mRNAs. Mol. Cell. Biol..

[106] Kim W.J., Back S.H., Kim V., Ryu I., Jang S.K. (2005). Sequestration of traf2 into stress granules interrupts tumor necrosis factor signaling under stress conditions. Mol. Cell. Biol..

[107] Kroschwald S., Maharana S., Mateju D., Malinovska L., Nuske E., Poser I., Richter D., Alberti S. (2015). Promiscuous interactions and protein disaggregases determine the material state of stress-inducible rnp granules. eLife.

[108] Gilks N., Kedersha N., Ayodele M., Shen L., Stoecklin G., Dember L.M., Anderson P. (2004). Stress granule assembly is mediated by prion-like aggregation of TIA-1. Mol. Biol. Cell.

[109] Panas M.D., Varjak M., Lulla A., Eng K.E., Merits A., Karlsson Hedestam G.B., McInerney G.M. (2012). Sequestration of G3BP coupled with efficient translation inhibits stress granules in semliki forest virus infection. Mol. Biol. Cell.

[110] Katoh H., Okamoto T., Fukuhara T., Kambara H., Morita E., Mori Y., Kamitani W., Matsuura Y. (2013). Japanese encephalitis virus core protein inhibits stress granule formation through an interaction with caprin-1 and facilitates viral propagation. J. Virol..

[111] Fros J.J., Domeradzka N.E., Baggen J., Geertsema C., Flipse J., Vlak J.M., Pijlman G.P. (2012). Chikungunya virus nsp3 blocks stress granule assembly by recruitment of G3BP into cytoplasmic foci. J. Virol..

[112] White J.P., Lloyd R.E. (2011). Poliovirus unlinks TIA1 aggregation and mRNA stress granule formation. J. Virol..

[113] Piotrowska J., Hansen S.J., Park N., Jamka K., Sarnow P., Gustin K.E. (2010). Stable formation of compositionally unique stress granules in virus-infected cells. J. Virol..

[114] Mok B.W., Song W., Wang P., Tai H., Chen Y., Zheng M., Wen X., Lau S.Y., Wu W.L., Matsumoto K. (2012). The ns1 protein of influenza a virus interacts with cellular processing bodies and stress granules through RNA-associated protein 55 (rap55) during virus infection. J. Virol..

[115] Onomoto K., Jogi M., Yoo J.S., Narita R., Morimoto S., Takemura A., Sambhara S., Kawaguchi A., Osari S., Nagata K. (2012). Critical role of an antiviral stress granule containing rig-i and PKR in viral detection and innate immunity. PLoS ONE.

[116] Simpson-Holley M., Kedersha N., Dower K., Rubins K.H., Anderson P., Hensley L.E., Connor J.H. (2011). Formation of antiviral cytoplasmic granules during orthopoxvirus infection. J. Virol..

[117] Liu J., Wennier S., Zhang L., McFadden G. (2011). M062 is a host range factor essential for myxoma virus pathogenesis and functions as an antagonist of host SAMD9 in human cells. J. Virol..

[118] Zhang P., Langland J.O., Jacobs B.L., Samuel C.E. (2009). Protein kinase PKR-dependent activation of mitogen-activated protein kinases occurs through mitochondrial adapter ips-1 and is antagonized by vaccinia virus e3l. J. Virol..

[119] Lemos de Matos A., Liu J., McFadden G., Esteves P.J. (2013). Evolution and divergence of the mammalian SAMD9/SAMD9l gene family. BMC Evol. Biol..

[120] Meng X., Chao J., Xiang Y. (2008). Identification from diverse mammalian poxviruses of host-range regulatory genes functioning equivalently to vaccinia virus C7l. Virology.

[121] Liu J., Rothenburg S., McFadden G. (2012). The poxvirus C7l host range factor superfamily. Curr. Opin. Virol..

[122] Perkus M.E., Goebel S.J., Davis S.W., Johnson G.P., Limbach K., Norton E.K., Paoletti E. (1990). Vaccinia virus host range genes. Virology.

[123] Meng X., Krumm B., Li Y., Deng J., Xiang Y. (2015). Structural basis for antagonizing a host restriction factor by C7 family of poxvirus host-range proteins. Proc. Natl. Acad. Sci. USA.

[124] Sivan G., Ormanoglu P., Buehler E.C., Martin S.E., Moss B. (2015). Identification of restriction factors by human genome-wide RNA interference screening of viral host range mutants exemplified by discovery of SAMD9 and wdr6 as inhibitors of the vaccinia virus k1l-C7l- mutant. mBio.

[125] Ng C.S., Jogi M., Yoo J.S., Onomoto K., Koike S., Iwasaki T., Yoneyama M., Kato H., Fujita T. (2013). Encephalomyocarditis virus disrupts stress granules, the critical platform for triggering antiviral innate immune responses. J. Virol..

[126] Meng X., Schoggins J., Rose L., Cao J., Ploss A., Rice C.M., Xiang Y. (2012). C7l family of poxvirus host range genes inhibits antiviral activities induced by type i interferons and interferon regulatory factor 1. J. Virol..

[127] Reineke L.C., Lloyd R.E. (2015). The stress granule protein G3BP1 recruits protein kinase r to promote multiple innate immune antiviral responses. J. Virol..

[128] Langereis M.A., Feng Q., van Kuppeveld F.J. (2013). Mda5 localizes to stress granules, but this localization is not required for the induction of type i interferon. J. Virol..

[129] Iordanov M.S., Wong J., Bell J.C., Magun B.E. (2001). Activation of nf-kappab by double-stranded RNA (dsRNA) in the absence of protein kinase r and RNAse l demonstrates the existence of two separate dsRNA-triggered antiviral programs. Mol. Cell. Biol..

[130] Khaperskyy D.A., Hatchette T.F., McCormick C. (2012). Influenza a virus inhibits cytoplasmic stress granule formation. FASEB J..

[131] Hato S.V., Ricour C., Schulte B.M., Lanke K.H., de Bruijni M., Zoll J., Melchers W.J., Michiels T., van Kuppeveld F.J. (2007). The mengovirus leader protein blocks interferon-alpha/beta gene transcription and inhibits activation of interferon regulatory factor 3. Cell. Microbiol..

[132] Yoshida A., Kawabata R., Honda T., Tomonaga K., Sakaguchi T., Irie T. (2015). Ifn-beta-inducing, unusual viral RNA species produced by paramyxovirus infection accumulated into distinct cytoplasmic structures in an RNA-type-dependent manner. Front. Microbiol..

[133] Peters N.E., Ferguson B.J., Mazzon M., Fahy A.S., Krysztofinska E., Arribas-Bosacoma R., Pearl L.H., Ren H., Smith G.L. (2013). A mechanism for the inhibition of DNA-pk-mediated DNA sensing by a virus. PLoS Pathog..

[134] Lam E., Stein S., Falck-Pedersen E. (2014). Adenovirus detection by the cgas/sting/tbk1 DNA sensing cascade. J. Virol..

[135] Unterholzner L., Keating S.E., Baran M., Horan K.A., Jensen S.B., Sharma S., Sirois C.M., Jin T., Latz E., Xiao T.S. (2010). Ifi16 is an innate immune sensor for intracellular DNA. Nat. Immunol..

[136] Jakobsen M.R., Bak R.O., Andersen A., Berg R.K., Jensen S.B., Tengchuan J., Laustsen A., Hansen K., Ostergaard L., Fitzgerald K.A. (2013). Ifi16 senses DNA forms of the lentiviral replication cycle and controls HIV-1 replication. Proc. Natl. Acad. Sci. USA.

[137] Doitsh G., Galloway N.L., Geng X., Yang Z., Monroe K.M., Zepeda O., Hunt P.W., Hatano H., Sowinski S., Munoz-Arias I. (2014). Cell death by pyroptosis drives cd4 t-cell depletion in HIV-1 infection. Nature.

[138] Monroe K.M., Yang Z., Johnson J.R., Geng X., Doitsh G., Krogan N.J., Greene W.C. (2014). Ifi16 DNA sensor is required for death of lymphoid cd4 t cells abortively infected with HIV. Science.

[139] Ansari M.A., Dutta S., Veettil M.V., Dutta D., Iqbal J., Kumar B., Roy A., Chikoti L., Singh V.V., Chandran B. (2015). Herpesvirus genome recognition induced acetylation of nuclear ifi16 is essential for its cytoplasmic translocation, inflammasome and ifn-beta responses. PLoS Pathog..

[140] Munoz-Arias I., Doitsh G., Yang Z., Sowinski S., Ruelas D., Greene W.C. (2015). Blood-derived cd4 t cells naturally resist pyroptosis during abortive HIV-1 infection. Cell Host Microbe.

[141] Orzalli M.H., Broekema N.M., Diner B.A., Hancks D.C., Elde N.C., Cristea I.M., Knipe D.M. (2015). Cgas-mediated stabilization of ifi16 promotes innate signaling during herpes simplex virus infection. Proc. Natl. Acad. Sci. USA.

[142] Horan K.A., Hansen K., Jakobsen M.R., Holm C.K., Soby S., Unterholzner L., Thompson M., West J.A., Iversen M.B., Rasmussen S.B. (2013). Proteasomal degradation of herpes simplex virus capsids in macrophages releases DNA to the cytosol for recognition by DNA sensors. J. Immunol..

[143] Veeranki S., Choubey D. (2012). Interferon-inducible p200-family protein ifi16, an innate immune sensor for cytosolic and nuclear double-stranded DNA: Regulation of subcellular localization. Mol. Immunol..

[144] Ishikawa H., Ma Z., Barber G.N. (2009). Sting regulates intracellular DNA-mediated, type i interferon-dependent innate immunity. Nature.

[145] Chien Y., Kim S., Bumeister R., Loo Y.M., Kwon S.W., Johnson C.L., Balakireva M.G., Romeo Y., Kopelovich L., Gale M. (2006). Ralb gtpase-mediated activation of the ikappab family kinase tbk1 couples innate immune signaling to tumor cell survival. Cell.

[146] Fons R.D., Bogert B.A., Hegde R.S. (2003). Substrate-specific function of the translocon-associated protein complex during translocation across the er membrane. J. Cell Biol..

[147] Ishikawa H., Barber G.N. (2008). Sting is an endoplasmic reticulum adaptor that facilitates innate immune signalling. Nature.

[148] Saitoh T., Fujita N., Hayashi T., Takahara K., Satoh T., Lee H., Matsunaga K., Kageyama S., Omori H., Noda T. (2009). ATG9A controls dsDNA-driven dynamic translocation of sting and the innate immune response. Proc. Natl. Acad. Sci. USA.

[149] Berg R.K., Rahbek S.H., Kofod-Olsen E., Holm C.K., Melchjorsen J., Jensen D.G., Hansen A.L., Jorgensen L.B., Ostergaard L., Tolstrup M. (2014). T cells detect intracellular DNA but fail to induce type i ifn responses: Implications for restriction of HIV replication. PLoS ONE.

[150] Stetson D.B., Medzhitov R. (2006). Recognition of cytosolic DNA activates an irf3-dependent innate immune response. Immunity.

[151] Ferguson B.J., Mansur D.S., Peters N.E., Ren H., Smith G.L. (2012). DNA-pk is a DNA sensor for irf-3-dependent innate immunity. eLife.

[152] Zhang Z., Yuan B., Bao M., Lu N., Kim T., Liu Y.J. (2011). The helicase ddx41 senses intracellular DNA mediated by the adaptor sting in dendritic cells. Nat. Immunol..

[153] Kondo T., Kobayashi J., Saitoh T., Maruyama K., Ishii K.J., Barber G.N., Komatsu K., Akira S., Kawai T. (2013). DNA damage sensor mre11 recognizes cytosolic double-stranded DNA and induces type i interferon by regulating sting trafficking. Proc. Natl. Acad. Sci. USA.

[154] Sun L., Wu J., Du F., Chen X., Chen Z.J. (2013). Cyclic gmp-amp synthase is a cytosolic DNA sensor that activates the type i interferon pathway. Science.

[155] Baptiste N., Prives C. (2004). P53 in the cytoplasm: A question of overkill?. Cell.

[156] Hornung V., Ablasser A., Charrel-Dennis M., Bauernfeind F., Horvath G., Caffrey D.R., Latz E., Fitzgerald K.A. (2009). Aim2 recognizes cytosolic dsDNA and forms a caspase-1-activating inflammasome with asc. Nature.

[157] Roberts T.L., Idris A., Dunn J.A., Kelly G.M., Burnton C.M., Hodgson S., Hardy L.L., Garceau V., Sweet M.J., Ross I.L. (2009). Hin-200 proteins regulate caspase activation in response to foreign cytoplasmic DNA. Science.

[158] Kis-Toth K., Szanto A., Thai T.H., Tsokos G.C. (2011). Cytosolic DNA-activated human dendritic cells are potent activators of the adaptive immune response. J. Immunol..

